# Factors Associated With Experiences of Harassment or Abuse Among Lesbian, Gay, Bisexual, Trans, Queer, and Asexual Young People With Disability in Australia

**DOI:** 10.1177/08862605231216690

**Published:** 2023-12-08

**Authors:** Natalie Amos, Adam O. Hill, Anthony Lyons, Christine Bigby, Marina Carman, Matthew Parsons, Adam Bourne

**Affiliations:** 1 La Trobe University, Bundoora, VIC, Australia; 2 St Luke’s International University, Tokyo, Japan; 3 Kirby Institute, UNSW Sydney, Sydney NSW, Australia

**Keywords:** disability, LGBT, abuse, assault, exclusion

## Abstract

Lesbian, gay, bisexual, trans, queer, and asexual (LGBTQA+) young people with disability are known to experience higher rates of harassment or abuse than LGBTQA+ young people without disability. This study focused on participants in Australia and identified factors associated with harassment or abuse among LGBTQA+ adolescents and young adults who reported a disability as well as associations with mental health outcomes. Analyses were conducted from a national survey that included 2,500 LGBTQA+ people who reported a disability and were aged 14 to 21 years. Measures included experiences in the past 12 months of verbal and physical harassment or abuse due to one’s sexual orientation or gender identity, sexual harassment or abuse, mental health, suicidality, and sociodemographic traits. Overall, 48.4% of participants with disability reported experiencing verbal harassment or abuse, 12.4% physical harassment or abuse, and 29.7% sexual assault or harassment. In multivariable regression analyses, verbal harassment or abuse was significantly more likely among trans men, participants with an intellectual disability, and those who were “out” to most or all of their family. Physical harassment or abuse was significantly more likely among participants with a physical or sensory disability. Sexual harassment or abuse was significantly more likely among trans women and participants with a physical or sensory disability. Participants who experienced harassment or abuse were also significantly more likely to have attempted suicide in the past 12 months. These findings will assist policymakers and practitioners in identifying contexts linked to a heightened risk of abuse among LGBTQA+ young people with disability and further underscore an immediate need to address and prevent harm in this population.

Lesbian, gay, bisexual, trans, queer, and asexual (LGBTQA+) young people can face a range of challenges related to stigma and discrimination (Bucchianeri et al., 2016). This population is also particularly at risk of concerns related to mental health and well-being (Hill et al., 2021) and can experience significant stress in the course of disclosing or “coming out” with regard to their sexual orientation or gender identity (Baams et al., 2015; Pollitt et al., 2017). In many instances, this can result in experiencing harassment or abuse from others, such as bullying at school (Eisenberg et al., 2015; Henderson, 2016). However, the broader population of LGBTQA+ young people is diverse, and some subpopulations may be additionally at risk based on other individual characteristics. LGBTQA+ young people with disability comprise one such group.

In this article, we refer to young people as adolescents and young adults up to the age of 21 years and specifically focus on the 14- to 21-year-old group. Relatively little research has focused on LGBTQA+ young people with disability, particularly regarding experiences of harassment or abuse in relation to their sexual orientation or gender identity. Studies, both nationally and globally, that have included LGBTQA+ young people with a disability indicate that they experience high rates of harassment or abuse (Bucchianeri et al., 2016; Leonard & Mann, 2018; McGee, 2014). There is also some evidence that this group can feel isolated or excluded from both LGBTQA+ and disability communities (Vaughn et al., 2015). This is potentially due to a form of dual marginalization where they feel marginalized by their disability in LGBTQA+ circles and also marginalized due to their sexual orientation or gender identity in disability circles (O’Shea et al., 2020; Wilson et al., 2018). In a recent Australian focus group study of LGBTIQA+ people with disability, participants expressed difficulties in managing multiple identities within the healthcare setting, with difficulties disclosing LGBTIQ+ identities within the disability sector and feelings of exclusion from LGBTIQ+ spaces due to their disability (O’Shea et al., 2020). A meta-analysis involving several countries also found that LGBT youth with disabilities supported by social service providers, special education programs, or supported living facilities reported being prohibited or restricted from expressing/discussing their LGBT identities (Duke, 2011). It is also important to note, as detailed by findings from the Australian Institute of Health and Welfare, that experiences of abuse and discrimination may not be uniformly felt among people with diverse forms of disability (Australian Institute of Health and Welfare, 2020).

Research conducted in the United States also found that young LGBQ students with disabilities who reported experiences of victimization had the highest rates of suicidal ideation compared to their heterosexual peers without a disability (King et al., 2018). It is especially important to gain a greater understanding of demographic and psychosocial predictors of experiences of harassment or abuse in relation to one’s sexual orientation or gender identity. Such information would be useful for understanding and further exploring potential drivers of these experiences as well as identifying subgroups that may be particularly impacted. This would be especially beneficial to service providers and policymakers to inform efforts for preventing and addressing experiences of violence or abuse. To our knowledge, little research on this is available. Some studies have documented abuse against LGBTQA+ young people with disability in some settings such as school and university (Bucchianeri et al., 2016; King et al., 2018; Lund & Ross, 2021) and among caretakers (Duke, 2011). LGBTQA+ people and those with a disability are diverse groups who may differentially experience harassment or abuse in line with other axes of inequality (e.g., age, ethnicity, area of residence etc.). However, most research to date has been insufficiently nuanced, or lacked sufficient sample size, to explore the experiences of harassment or abuse among LGBTQA+ young people with disability and address the significant level of unmet intervention need for this group.

In this study, we report on the analysis of a subset of data drawn from an Australian national survey of LGBTQA+ young people in Australia aged 14 to 21 years. The data included LGBTQA+ young people from the main survey who identified as having a disability. We examined a range of potential demographic and psychosocial predictors of reported experiences of harassment or abuse based on their sexual orientation or gender identity over the previous 12 months. Specifically, our aim was to identify statistically significant predictors to help inform a greater understanding of these experiences and sites for interventions. We focused on three types of harassment or abuse, including verbal, physical, and sexual to provide broad findings across a range of different challenges. We also examine how such experiences are associated with mental health, specifically the experience of psychological distress, suicidal ideation and/or attempting suicide.

## Method

### Sample and Procedure

The sample for this study was drawn from the *Writing Themselves In 4* survey, which was conducted in late 2019 and prior to the COVID-19 pandemic. This was a large, national, cross-sectional survey of the health and well-being of LGBTQA+ young people in Australia aged 14 to 21 years. Participants were recruited via Facebook and Instagram advertising, as well as through promotional material circulated by LGBTIQ youth services and LGBTIQ community organizations. Prior to starting the survey, participants read the background information for the study, gave informed consent, and were also informed that their responses would be confidential and anonymous and that they could skip any questions they did not wish to answer. In total, 6,418 participants completed the survey. Of this group, 2,500 reported having one or more forms of disability (outlined below) and therefore comprised the sample for analysis in this article.

### Materials

Items from the survey that were included in the analyses for this article were:

#### Disability

Assessment of disability relied on self-reported identification of having a disability, defined as “experiencing neurodiversity/autism, or having a long-term physical or mental health condition. Long-term health conditions could include things like epilepsy, mental health conditions, and speech or sensory impairments. A disability could include things such as the loss of—or difficulty using—a body part, or difficulty managing everyday activities.” Participants who selected “yes” were then asked to indicate the type of disability or long-term health condition from the following seven choices: “autism or neurodiversity,” “intellectual disability,” “physical disability,” “sensory disability,” “mental health condition,” “acquired brain injury,” and “other disability.” Disability categories presented and analyzed in this article were created in consultation with a Disability Advisory Board of community experts with disability.

#### Harassment/Abuse Variables

Participants were asked if they had experienced any of the following forms of harassment or abuse in the past 12 months based on their sexual orientation or gender identity: verbal (e.g., called names or threatened); physical (e.g., shoved, punched, or injured with a weapon); and sexual (e.g., unwanted touching, sexual remarks, sexual messages, or being forced to perform any unwanted sexual act). Separate variables were created for the three types of harassment or abuse to indicate whether participants reported each of these (yes/no) in the past 12 months.

#### Mental Health

A range of survey items were used to assess mental health, suicidal ideation, and suicide attempts. The K10 Psychological Distress Scale was used to assess overall mental health and is validated among young people in Australia (Smout, 2019). It includes items that cover symptoms of anxiety and depression in the past 4 weeks, such as “so nervous that nothing could calm you down” and “depressed.” Participants responded on a five-point scale from “none of the time” to “all of the time.” Total scores were between 10 and 50, which were then classified into low (10–15), moderate (16–21), high (22–29), or very high (30–50) levels of psychological distress, according to commonly used criteria (Australian Bureau of Statistics, 2012). To facilitate analysis, these were further grouped into a dichotomous variable with categories of low/moderate and high/very high.

Participants were asked, “have you experienced thoughts about suicide, wanting to die or about ending your life” (suicidal ideation) and also “have you attempted suicide or to end your life.” For both questions, response options included “no,” “yes, in the past 12 months,” “yes, more than 12 months ago,” and “prefer not to answer.” To focus on recent experiences, dichotomous variables were computed to indicate whether or not participants reported having experienced suicidal ideation or had attempted suicide in the past 12 months.

#### Sociodemographics

Questions on sociodemographics included age, area of residence (city—inner suburban, city—outer suburban, regional, rural), education in the past 12 months, engagement in paid employment, and country of birth. To categorize gender, participants who were selected from a list of gender identities were then grouped into five categories: cisgender woman, cisgender man, trans woman, trans man, and non-binary. Cisgender refers to those whose sex on their original birth certificate matches their current gender identity. Participants were categorized as trans women if they were assumed male at birth and chose only “woman,” “trans woman,” or a related term. Participants were categorized as trans men if they were assumed female at birth and chose only “man,” “trans man,” or a related term. Participants were categorized as non-binary if they selected a gender that was not binary (e.g., genderfluid) or were unable to choose a single gender identity. To categorize sexual orientation, participants chose from a list of sexual identities along with additional options of “prefer not to have a label,” “don’t know,” and “something different.” Participants were grouped into one of seven categories; lesbian, gay, bisexual, pansexual, queer, asexual, and something different.

Participants were also asked to what extent they had disclosed, or were “out,” to their sexual or gender identity to family. A variable was computed with four categories, including being out to no family members, a few or some family members, most or all family members, or not applicable. The “not applicable” option was provided for any participants who did not have family or somehow felt that the question was not relevant to their circumstances.

### Statistical Analysis

Numbers and percentages were first computed for all variables prior to conducting a sequence of logistic regressions. These regressions focused on the three outcome variables of verbal, physical, and sexual harassment or abuse in the past 12 months. For each outcome variable, univariable regressions were first conducted for each predictor variable. This was followed by a multivariable regression to identify significant independent predictor variables. Independent variables comprised all other study variables, including variables to indicate different types of disability as well as the mental health and sociodemographic variables. Participants were excluded from an analysis where they had missing data on a variable in that particular analysis. Collinearity tests of the study variables were conducted, which revealed no issues with multicollinearity, with all variance inflation factors <5. STATA 16 (StataCorp, College Station, TX, USA) was used for all analyses.

## Results

### Descriptive Statistics

Table 1 displays numbers and percentages for all variables. Almost one-third (30.9%) of participants with disability identified as bisexual. Almost half (47.8%) were cisgender women and 27.8% were non-binary. A majority (52.9%) were aged 14 to 17 years, attending secondary school (53.6%), engaged in some form of paid employment (58.1%), born in Australia (92.4%), and living in the outer suburban areas of capital cities (57.0%). A large majority reported being out to some or all members of their family, with 20.2% not out to any family member. Regarding type of disability, approximately one-third (34.6%) reported autism or neurodiversity, 13.9% intellectual disability, 28.8% physical or sensory disability, 88.2% a mental health condition, and 5.7% an acquired brain injury or another disability that was not listed. Most participants reported having experienced suicidal ideation in the past 12 months (73.2%) or high/very high psychological distress (92.0%), while 16.8% reported having attempted suicide in the past 12 months. Overall, 48.4% reported experiencing verbal harassment or abuse, 12.4% physical harassment or abuse, and 29.7% sexual harassment or abuse in the past 12 months.

**Table 1.** Sample Characteristics.

Sample Characteristics	*n*	%
Age		
14–17	1,323	52.9
18–21	1,177	47.1
Sexual orientation		
Lesbian	342	13.7
Gay	280	11.2
Bisexual	771	30.9
Pansexual	354	14.2
Queer	267	10.7
Asexual	133	5.3
Something else	350	14.0
Gender		
Cisgender man	303	12.6
Cisgender woman	1,150	47.8
Trans man	239	9.9
Trans woman	46	1.9
Non-binary	668	27.8
Education		
Secondary school (high school)	1,259	53.6
University	627	26.7
Nonuniversity tertiary	257	10.9
Other	207	8.8
Employment		
No	1,047	41.9
Yes	1,451	58.1
Country of birth		
Australia born	2,307	92.4
Other English-speaking country	128	5.1
Non-English-speaking country	63	2.5
Residential location		
Capital city, inner suburban	167	6.7
Capital city, outer suburban	1,423	57.0
Regional city or town	620	24.8
Rural/remote	288	11.5
Out to family		
No	503	20.2
A few/some	1,177	47.3
Most/all	760	30.6
Not applicable	48	1.9
Autism or neurodiverse		
No	1,634	65.4
Yes	866	34.6
Intellectual disability		
No	2,153	86.1
Yes	347	13.9
Physical or sensory disability		
No	1,781	71.2
Yes	719	28.8
Mental health condition		
No	294	11.8
Yes	2,206	88.2
Acquired brain injury or another disability that was not listed		
No	2,358	94.3
Yes	142	5.7
Recent suicidal ideation		
No	642	26.8
Yes	1,753	73.2
Recent suicide attempt		
No	1,914	83.2
Yes	387	16.8
Psychological distress		
Low or moderate	199	8.0
High or very high	2,287	92.0
Verbal harassment or abuse in past 12 months		
No	1,246	51.6
Yes	1,170	48.4
Physical harassment or abuse in past 12 months		
No	1,880	87.7
Yes	265	12.4
Sexual harassment or abuse in past 12 months		
No	1,559	70.3
Yes	660	29.7

### Factors Associated With Verbal Harassment or Abuse

Table 2 displays the regression results for experiences of harassment or verbal abuse. A range of significant predictors were identified in the multivariable analysis. Participants with disability were significantly more likely to report having experienced verbal harassment or abuse in the past 12 months if they were a trans man (Adjusted Odds Ratio; AOR = 1.72, 95% Confidence Interval; CI [1.10, 2.70], *p* = .018), were in paid employment (AOR = 1.28, 95% CI [1.05, 1.57], *p* = .017), were out to most or all of their family memebers (AOR = 1.35, 95% CI [1.02, 1.79], *p* = .039), or reported an intellectual disability (AOR = 1.62, 95% CI [1.20, 2.19], *p* = .002), or a mental health condition (AOR = 1.47, 95% CI [1.07, 2.03], *p* = .019). Participants were significantly less likely to report having experienced verbal harassment or abuse if they were a cisgender woman (AOR = 0.37, 95% CI [0.26, 0.53], *p* < .001). Participants who reported experiencing suicidal ideation in the past 12 months (AOR = 1.42, 95% CI [1.12, 1.80], *p* = .004) or attempted suicide in the past 12 months (AOR = 2.18, 95% CI [1.66, 2.87], *p* < .001), or had high/very high psychological distress in the past 4 weeks (AOR = 2.11, 95% CI [1.42, 3.13], *p* < .001) were also significantly more likely to have reported experiencing verbal harassment or abuse.

**Table 2.** Factors Associated With Experiencing Verbal Harassment or Abuse in the Past 12 Months Among Lesbian, Gay, Bisexual, Trans, Queer, and Asexual Young People With Disability (*n* = 1,965).

Independent variables	*n*	%	Odds Ratio (OR) [95% CI]	*p*-Value	AOR [95% CI]	*p*-Value
Age group (years)						
14–17	638	49.8	REF		REF	
18–21	532	46.9	0.89 [0.76, 1.04]	.150	0.81 [0.61, 1.09]	.165
Sexual orientation						
Gay	157	57.1	REF		REF	
Lesbian	167	49.6	0.74 [0.54, 1.02]	.063	1.17 [0.76, 1.78]	.473
Bisexual	300	41.0	0.52 [0.39, 0.69]	.000	0.75 [0.52, 1.08]	.124
Pansexual	183	53.4	0.86 [0.62, 1.18]	.353	0.86 [0.57, 1.31]	.492
Queer	147	56.5	0.98 [0.69, 1.38]	.897	0.99 [0.63, 1.56]	.978
Asexual	52	40.3	0.51 [0.33, 0.78]	.002	0.69 [0.40, 1.19]	.179
Something else	163	48.4	0.70 [0.51, 0.97]	.032	0.72 [0.48, 1.09]	.120
Gender						
Cisgender man	159	54.6	REF		REF	
Cisgender woman	393	35.3	0.45 [0.35, 0.59]	.000	0.37 [0.26, 0.53]	.000
Trans man	169	72.5	2.19 [1.52, 3.17]	.000	1.72 [1.10, 2.70]	.018
Trans woman	31	68.9	1.84 [0.94, 3.60]	.076	1.17 [0.56, 2.45]	.682
Non-binary	370	57.6	1.13 [0.85, 1.49]	.393	0.92 [0.63, 1.34]	.658
Education						
Secondary school (high school)	599	49.4	REF		REF	
University	278	46.1	0.88 [0.72, 1.07]	.188	0.98 [0.70, 1.37]	.904
Nonuniversity tertiary	125	50.2	1.03 [0.79, 1.36]	.814	0.91 [0.63, 1.31]	.598
Other	98	47.8	0.94 [0.70, 1.26]	.676	0.90 [0.62, 1.33]	.612
Employed in past 12 months						
No	478	47.2	REF		REF	
Yes	691	49.3	1.09 [0.92, 1.28]	.319	1.28 [1.05, 1.57]	.017
Country of birth						
Australia born	1,073	48.2	REF		REF	
Other English-speaking country	50	51.6	1.14 [0.76, 1.72]	.517	1.24 [0.74, 2.07]	.413
Non-English-speaking country	47	51.1	1.12 [0.74, 1.70]	.585	1.07 [0.63, 1.79]	.810
Residential location						
Capital city, inner suburban	75	48.1	REF		REF	
Capital city, outer suburban	666	48.1	1.00 [0.72, 1.39]	.998	0.99 [0.64, 1.52]	.953
Regional city or town	273	45.9	0.92 [0.64, 1.30]	.625	0.84 [0.53, 1.35]	.479
Rural/remote	155	55.8	1.36 [0.92, 2.02]	.125	1.12 [0.67, 1.87]	.667
Out to family						
No	191	39.4	REF		REF	
A few or some	548	48.1	1.42 [1.15, 1.77]	.001	1.25 [0.97, 1.62]	.083
Most or all	406	55.1	1.89 [1.50, 2.38]	.000	1.35 [1.02, 1.79]	.039
Not applicable	20	44.4	1.23 [0.67, 2.28]	.508	1.02 [0.44, 2.34]	.965
Autism or neurodiverse						
No	719	45.5	REF		REF	
Yes	451	53.9	1.40 [1.18, 1.65]	.000	1.17 [0.94, 1.45]	.164
Intellectual disability						
No	961	46.2	REF		REF	
Yes	209	62.0	1.90 [1.50, 2.41]	.000	1.62 [1.20, 2.19]	.002
Physical or sensory disability						
No	799	46.3	REF		REF	
Yes	371	53.8	1.35 [1.13, 1.61]	.001	1.25 [1.00, 1.56]	.052
Mental health condition						
No	114	41.0	REF		REF	
Yes	1,056	49.4	1.40 [1.09, 1.81]	.009	1.47 [1.07, 2.03]	.019
Acquired brain injury or another disability that was not listed						
No	1,110	48.7	REF		REF	
Yes	60	43.8	0.82 [0.58, 1.16]	.265	0.94 [0.62, 1.43]	.772
Recent suicidal ideation						
No	227	36.4	REF		REF	
Yes	900	53.1	1.98 [1.64, 2.39]	.000	1.42 [1.12, 1.80]	.004
Recent suicide attempt						
No	821	44.4	REF		REF	
Yes	256	67.4	2.58 [2.05, 3.26]	.000	2.18 [1.66, 2.87]	.000
Psychological distress						
Low or moderate	51	26.8	REF		REF	
High or very high	1,112	50.3	2.75 [1.98, 3.84]	.000	2.11 [1.42, 3.13]	.000

### Factors Associated With Physical Harassment or Abuse

Table 3 displays theregression results for experiences of physical harassment or abuse. In the multivariable analysis, participants with disability were significantly more likely to report having experienced physical harassment or abuse in the past 12 months if they reported a physical or sensory disability (AOR = 1.79, 95% CI [1.26, 2.55], *p* = .001), a mental health condition (AOR = 3.02, 95% CI [1.45, 6.29], *p* = .003), or either an acquired brain injury or another disability that was not listed (AOR = 2.23, 95% CI [1.25, 3.99], *p* = .007). They were significantly less likely to report having experienced physical harassment or abuse if they were a cisgender woman (AOR = 0.23, 95% CI [0.13, 0.40], *p* < .001) or were non-binary (AOR = 0.50, 95% CI [0.29, 0.86], *p* = .012). Participants who reported experiencing suicidal ideation in the past 12 months (AOR = 1.76, 95% CI [1.07, 2.90], *p* = .026) or attempted suicide in the past 12 months (AOR = 3.22, 95% CI [2.27, 4.58], *p* < .001) were also significantly more likely to have experienced physical harassment or abuse.

**Table 3.** Factors Associated With Experiencing Physical Harassment or Abuse in the Past 12 Months Among Lesbian, Gay, Bisexual, Trans, Queer, and Asexual Young People With Disability (*n* = 1,764).

Independent variables	*n*	%	OR [95% CI]	*p*-Value	AOR [95% CI]	*p*-Value
Age group (years)						
14–17	159	14.2	REF		REF	
18–21	106	10.3	0.70 [0.54, 0.90]	.007	0.85 [0.54, 1.33]	.474
Sexual orientation						
Gay	46	18.7	REF		REF	
Lesbian	35	12.0	0.59 [0.37, 0.95]	.031	0.92 [0.48, 1.75]	.794
Bisexual	60	9.2	0.44 [0.29, 0.66]	.000	0.82 [0.47, 1.43]	.493
Pansexual	49	16.1	0.83 [0.53, 1.30]	.416	1.06 [0.57, 1.97]	.844
Queer	34	15.1	0.77 [0.48, 1.26]	.301	0.84 [0.43, 1.63]	.603
Asexual	8	6.8	0.32 [0.15, 0.70]	.004	0.39 [0.13, 1.16]	.090
Something else	32	10.6	0.52 [0.32, 0.84]	.008	0.58 [0.32, 1.05]	.070
Gender						
Cisgender man	47	18.4	REF		REF	
Cisgender woman	71	7.2	0.35 [0.23, 0.51]	.000	0.23 [0.13, 0.40]	.000
Trans man	39	18.9	1.03 [0.65, 1.66]	.891	0.63 [0.34, 1.17]	.145
Trans woman	8	21.1	1.18 [0.51, 2.74]	.700	0.84 [0.33, 2.14]	.708
Non-binary	84	14.3	0.74 [0.50, 1.09]	.131	0.50 [0.29, 0.86]	.012
Education						
Secondary school (high school)	146	13.7	REF		REF	
University	48	8.8	0.60 [0.43, 0.85]	.004	0.64 [0.37, 1.11]	.115
Nonuniversity tertiary	29	12.9	0.93 [0.61, 1.42]	.737	0.74 [0.42, 1.31]	.303
Other	24	13.6	0.99 [0.62, 1.57]	.950	0.86 [0.47, 1.56]	.616
Employed in past 12 months						
No	106	11.9	REF		REF	
Yes	158	12.6	1.07 [0.82, 1.39]	.627	1.28 [0.92, 1.77]	.144
Country of birth						
Australia born	243	12.3	REF		REF	
Other English-speaking country	12	14.1	1.18 [0.63, 2.20]	.613	1.32 [0.60, 2.95]	.491
Non-English-speaking country	10	12.5	1.02 [0.52, 2.01]	.952	1.10 [0.52, 2.33]	.812
Residential location						
Capital city, inner suburban	17	12.3	REF		REF	
Capital city, outer suburban	139	11.3	0.91 [0.53, 1.55]	.719	0.94 [0.47, 1.88]	.863
Regional city or town	62	11.7	0.94 [0.53, 1.67]	.841	0.80 [0.37, 1.70]	.557
Rural/remote	46	18.8	1.65 [0.90, 3.00]	.104	1.43 [0.65, 3.16]	.371
Out to family						
No	43	10.0	REF		REF	
A few or some	119	11.7	1.20 [0.83, 1.73]	.340	1.06 [0.69, 1.64]	.778
Most or all	95	14.4	1.52 [1.04, 2.23]	.032	1.02 [0.64, 1.63]	.938
Not applicable	6	17.1	1.86 [0.73, 4.74]	.192	1.71 [0.45, 6.50]	.431
Autism or neurodiverse						
No	154	11.1	REF		REF	
Yes	111	14.6	1.37 [1.06, 1.79]	.018	1.17 [0.83, 1.65]	.375
Intellectual disability						
No	201	10.9	REF		REF	
Yes	64	21.8	2.30 [1.68, 3.14]	.000	1.53 [0.99, 2.35]	0.053
Physical or sensory disability						
No	164	10.7	REF		REF	
Yes	101	16.4	1.64 [1.25, 2.14]	.000	1.79 [1.26, 2.55]	.001
Mental health condition						
No	16	6.4	REF		REF	
Yes	249	13.2	2.22 [1.32, 3.75]	.003	3.02 [1.45, 6.29]	.003
Acquired brain injury or another disability that was not listed						
No	245	12.1	REF		REF	
Yes	20	16.3	1.41 [0.86, 2.32]	.177	2.23 [1.25, 3.99]	.007
Recent suicidal ideation						
No	33	5.9	REF		REF	
Yes	222	14.7	2.73 [1.87, 4.00]	.000	1.76 [1.07, 2.90]	.026
Recent suicide attempt						
No	151	9.1	REF		REF	
Yes	92	28.1	3.94 [2.93, 5.28]	.000	3.22 [2.27, 4.58]	.000
Psychological distress						
Low or moderate	11	6.1	REF		REF	
High or very high	253	12.9	2.28 [1.22, 4.26]	.010	1.31 [0.55, 3.12]	.538

### Factors Associated With Sexual Harassment or Abuse

Table 4 displays the regression results for experiences of sexual harassment or abuse. In the multivariable analysis, participants with disability were significantly more likely to report having experienced sexual harassment or abuse in the past 12 months if they were a trans woman (AOR = 2.85, 95% CI [1.31, 6.19], *p* = .008), were in paid employment (AOR = 1.66, 95% CI [1.32, 2.09], *p* < .001), or reported a physical or sensory disability (AOR = 1.29, 95% CI [1.01, 1.64], *p* = .043) or a mental health condition (AOR = 1.90, 95% CI [1.27, 2.84], *p* = .002). Participants were significantly less likely to report having experienced sexual harassment or abuse in the past 12 months if they were living outside the inner areas of capital cities, including outer suburban (AOR = 0.56, 95% CI [0.37, 0.85], *p* = .006), regional (AOR = 0.59, 95% CI [0.37, 0.92], *p* = .019), and rural or remote (AOR = 0.59, 95% CI [0.36, 0.98], *p* = .043) areas. Participants who reported having attempted suicide in the past 12 months (AOR = 2.59, 95% CI [1.97, 3.41], *p* < .001) were also significantly more likely to have reported experiencing sexual harassment or abuse.

**Table 4.** Factors Associated With Experiencing Sexual Harassment or Abuse in the Past 12 months Among Lesbian, Gay, Bisexual, Trans, Queer, and Asexual Young People With Disability (*n* = 1,827).

Independent variables	*n*	%	OR [95% CI]	*p*-Value	AOR [95% CI]	*p*-Value
Age group (years)						
14–17	315	27.4	REF		REF	
18–21	345	32.3	1.26 [1.05, 1.52]	.012	1.25 [0.91, 1.72]	.161
Sexual orientation						
Gay	76	30.2	REF		REF	
Lesbian	103	33.8	1.18 [0.82, 1.69]	.364	1.18 [0.74, 1.89]	.483
Bisexual	197	29.1	0.95 [0.69, 1.31]	.762	1.09 [0.72, 1.64]	.696
Pansexual	93	28.8	0.94 [0.65, 1.34]	.721	0.88 [0.55, 1.42]	.604
Queer	78	33.3	1.16 [0.79, 1.70]	.453	1.04 [0.64, 1.71]	.863
Asexual	24	20.5	0.60 [0.35, 1.01]	.054	0.87 [0.47, 1.60]	.647
Something else	88	28.5	0.92 [0.64, 1.33]	.664	0.98 [0.62, 1.56]	.946
Gender						
Cisgender man	75	28.6	REF		REF	
Cisgender woman	290	28.4	0.99 [0.73, 1.34]	.943	0.97 [0.65, 1.44]	.884
Trans man	63	29.9	1.06 [0.71, 1.58]	.770	0.96 [0.59, 1.56]	.863
Trans woman	22	55.0	3.05 [1.55, 6.00]	.001	2.85 [1.31, 6.19]	.008
Non-binary	185	30.6	1.10 [0.80, 1.51]	.565	1.05 [0.69, 1.60]	.817
Education						
Secondary school (high school)	300	27.4	REF		REF	
University	197	34.4	1.39 [1.12, 1.73]	.003	1.11 [0.78, 1.60]	.554
Nonuniversity tertiary	67	29.0	1.08 [0.79, 1.48]	.614	0.90 [0.60, 1.34]	.591
Other	55	30.2	1.15 [0.82, 1.62]	.427	1.14 [0.78, 1.67]	.499
Employed in past 12 months						
No	214	23.4	REF		REF	
Yes	445	34.2	1.71 [1.41, 2.06]	.000	1.66 [1.32, 2.09]	.000
Country of birth						
Australia born	606	29.6	REF		REF	
Other English-speaking country	26	29.9	1.01 [0.63, 1.62]	.955	0.87 [0.49, 1.53]	.621
Non-English-speaking country	28	32.9	1.17 [0.74, 1.85]	.510	0.88 [0.51, 1.53]	.652
Residential location						
Capital city, inner suburban	60	41.1	REF		REF	
Capital city, outer suburban	373	29.3	0.59 [0.42, 0.84]	.004	0.56 [0.37, 0.85]	.006
Regional city or town	150	27.5	0.54 [0.37, 0.79]	.002	0.59 [0.37, 0.92]	.019
Rural/remote	76	29.9	0.61 [0.40, 0.94]	.024	0.59 [0.36, 0.98]	.043
Out to family						
No	135	30.1	REF		REF	
A few or some	286	27.3	0.87 [0.68, 1.11]	.274	0.81 [0.61, 1.07]	.142
Most or all	221	32.6	1.13 [0.87, 1.46]	.363	0.92 [0.67, 1.25]	.592
Not applicable	15	39.5	1.52 [0.77, 3.00]	.231	0.93 [0.39, 2.19]	.863
Autism or neurodiverse						
No	411	28.6	REF		REF	
Yes	249	31.8	1.16 [0.96, 1.40]	.118	1.21 [0.96, 1.54]	.108
Intellectual disability						
No	557	29.0	REF		REF	
Yes	103	34.2	1.27 [0.98, 1.64]	.068	1.14 [0.83, 1.58]	.421
Physical or sensory disability						
No	457	28.8	REF		REF	
Yes	203	32.0	1.16 [0.95, 1.42]	.138	1.29 [1.01, 1.64]	.043
Mental health condition						
No	48	18.7	REF		REF	
Yes	612	31.2	1.97 [1.42, 2.74]	.000	1.90 [1.27, 2.84]	.002
Acquired brain injury or another disability that was not listed						
No	621	29.7	REF		REF	
Yes	39	30.5	1.04 [0.70, 1.53]	.853	1.18 [0.75, 1.86]	.464
Recent suicidal ideation						
No	135	23.6	REF		REF	
Yes	504	32.2	1.54 [1.23, 1.92]	.000	1.07 [0.82, 1.38]	.637
Recent suicide attempt						
No	449	26.1	REF		REF	
Yes	162	47.0	2.51 [1.98, 3.18]	.000	2.59 [1.97, 3.41]	.000
Psychological distress						
Low or moderate	36	19.8	REF		REF	
High or very high	619	30.6	1.78 [1.22, 2.60]	.003	1.42 [0.91, 2.21]	.122

## Discussion

Large proportions of LGBTQA+ participants with disability aged 14 to 21 years reported experiencing verbal (48.4%), physical (12.4%), or sexual harassment or abuse (29.7%) based on their sexual orientation or gender identity in the past 12 months. These were higher than the proportions reported in the sample of participants without disability in the *Writing Themselves In 4* survey, in which 34.7% of those aged 14 to 21 years reported verbal harassment or abuse, 7.5% physical harassment or abuse, and 18.5% sexual harassment or abuse in the past 12 months (Hill et al., 2022). Thus, LGBTQA+ young people with disability appear to be especially at risk of experiencing several forms of harassment or abuse based on their sexual orientation or gender identity, suggesting a need for specific targeted preventative strategies to prevent experiences of harassment and abuse among this specific population.

This study examined a range of individual factors that may be useful in identifying potential drivers for experiences of harassment or abuse as well as subgroups that may be at additional risk. In general, trans women and trans men were more likely to report some form of harassment or abuse in the past 12 months based on their sexual orientation or gender identity, while cisgender women were generally less likely than other groups. It is often the case that trans people report higher frequencies of discrimination, harassment, and abuse, which is likely to arise from higher levels of stigma and nonacceptance from others across a range of settings (Butler et al., 2019; McDermott et al., 2018).

In the multivariable analyses, experiences of harassment or abuse also varied by the type of disability. This included a greater likelihood of verbal harassment or abuse for those with intellectual disability, physical and sexual harassment or abuse for those with physical or sensory disability, and all three forms of harassment or abuse for those with some type of mental health condition. These findings, along with a greater likelihood of some forms of abuse depending on where people lived and whether they were engaged in paid employment, suggest that individual circumstances also need to be taken into account when assessing risk or targeting support. Explaining these findings will require further research. While this research provides broad patterns, it would be especially beneficial for future studies to look closely at specific contexts in which abuse occurs, particularly in relation to type of disability. Qualitative research would be valuable in providing in-depth knowledge of different circumstances and settings in which experiences of abuse occur as well as how those experiences are addressed and the types of support available (if any) to victims.

Participants with disability who reported having experienced abuse in the past 12 months were also significantly more likely to report suicidal ideation, attempted suicide, and/or high levels of psychological distress. This finding further underscores the serious impact of abuse on the lives of victims. LGBTQA+ young people are known to have disproportionately high rates of mental health challenges, largely owing to the impact of stigma and discrimination (Flynn et al., 2016; Hatzenbuehler et al., 2010; Hill et al., 2021). This study points to experiences of abuse as potentially major factors in mental health outcomes among those with disability. It also suggests a need for not only preventing and addressing such experiences but also for providing significant mental health support where these experiences are known to have occurred.

Overall, findings from this study highlight an urgent need to improve the lives of LGBTQA+ young people with disability. A range of organizations, including health, social, and disability services as well as educational settings and workplaces, can assist in creating positive change. Ensuring that organizations are fully inclusive of not only people with disabilities but also people of diverse sexualities and genders would be important. This includes enabling safe spaces where young people are free from the negative impact of stigma and feel valued and respected. Unfortunately, the limited research to date suggests that disability services struggle to be responsive to the needs of LGBTQA+ young people with disabilities (Smith et al., 2022). Making programs available to organizations to ensure they meet standards of inclusivity and safety, including staff training, may be especially beneficial. Governments and policymakers can also play a role by ensuring standards are met across health and educational services as well as devising initiatives or programs to raise awareness and prevent abuse toward LGBTQA+ young people with disabilities.

There were some limitations to this study. First, the survey was cross-sectional, so we cannot be certain of the directions of causality between the predictor and outcome variables without follow-up longitudinal research. Second, it is not known how representative the sample was of the broader population of LGBTQA+ young people with disability. The sample was large and there was diversity across a range of demographic variables, which were also controlled for in the multivariable analyses. However, it is possible that some people with disability were not able to complete the survey. For example, some of those with intellectual disability might not have completed the survey due to challenges in doing so. Further research is required that involves different sampling and recruitment strategies. This survey was ultimately aimed more broadly toward a general LGBTQA+ young adult population, and research that is specifically designed for those with disability would be valuable in future. For now, our findings can be regarded as a starting point, with further research needed to corroborate these as well as seek further knowledge to explain and contextualize specific findings, such as with the use of in-depth qualitative research.

In all, this study involved a large sample of LGBTQA+ young people with disability that examined experiences of verbal, physical, and sexual abuse based on their sexual orientation or gender identity. A large proportion of participants reported experiencing these forms of abuse in the past 12 months. These findings highlight an urgent need for preventing and addressing experiences of abuse in this population. A range of potential circumstances or subgroups, such as types of disability and different genders, were also identified as additional predictors for reporting experiences of abuse. Although further research is required, these findings are likely to be useful to policymakers, services, educational providers, and workplaces in seeking ways to ensure safe, respectful, and supportive environments for LGBTQA+ young people with disability.
